# 

**Published:** 1910-04-16

**Authors:** 


### Epistaxis.

Physiologically the means of stopping such hæmorrhage is by raising the arms above the head. This causes contraction of the vessels of the head and neck, and may even induce a feeling of faintness. Of local measures adrenalin solution is the most effectual, but the nose must first be cleared of clots and then the solution applied by means of wool on the end of a probe.

### Nasal Obstruction and the Chest.

In the earlier stages of adenoids children will still breathe through the nose during sleep, the open mouth being used occasionally for a deep inspiration. It is this attempt at nasal breathing which is so mischievous in producing deformity of the chest. The thoracic apparatus is engaged in drawing air with great difficulty through the obstructed nasal passages, and as a result the yielding anterior chest-wall becomes permanently altered in shape. Mothers should be taught to watch the chests of their sleeping children, and not wait for the arrival of deformity.—*Mr. E. G. Waggett.*

### Appointments.

The Leeds General Infirmary.—Mr. G. Constable Hayes, F.R.C.S., has been appointed surgeon, with charge of ophthalmic out-patients; Mr. L. R. Braithwaite, F.R.C.S., third assistant surgeon; and Mr. J. A. Coupland, F.R.C.S., fourth assistant surgeon.

### GRADUATES' MEDICAL SCHOOLS.

DIARY FOR THE WEEK, APRIL 18 to APRIL 23.

### MEDICAL GRADUATES' COLLEGE AND POLYCLINIC, 22 Chenies Street, W.C.

At 4 p.m.—**April 18,** Dr. S. E. Dore, **Clinique (skin).**
At 5.15 p.m.—**April 18,** Dr. C. O. Hawthorne, **Mind Blindness.**
At 4 p.m.—**April 19,** Dr. G. A. Sutherland, **Clinique (medical).**
At 5.15 p.m.—**April 19,** Dr. Scanes Spicer, **The Modern Treatment of Hay Fever.**
At 4 p.m.—**April 20,** Mr. Arthur Edmunds, **Clinique (surgical).**
At 5.15 p.m.—**April 20,** Dr. W. H. B. Stoddart, **Neurasthenia and Psychasthenia.**
At 4 p.m.—**April 21,** Dr. Harry Campbell, **Clinique (medical).**
At 5.15 p.m.—**April 21,** Mr. Richard Lake, **The Diagnosis and Indications for Operation in Suppurative Disease of the Labyrinth.**
At 4 p.m.—**April 22,** Mr. H. L. Eason, **Clinique (eye).**

### CENTRAL LONDON THROAT AND EAR HOSPITAL, Gray's Inn Road, W.C.

At 3.45 p.m.—**April 19,** Mr. W. Stuart Low, **Accessory Sinuses.**
At 3.45 p.m.—**April 22,** Mr. W. Stuart Low, **Accessory Sinuses.**

### ROYAL SOCIETY OF MEDICINE, 15 Cavendish Square, W. (Temporary address during building.)

At 5.30 p.m.—**April 20, Balneological and Climatological Section:**
At 15 Cavendish Square, W.
*Adjourned discussion* on "Arthritis deformans" to be re-opened by Dr. A. E. Garrod.
*Paper*: Dr. Leigh Canney (Assouan): "The climate of Egypt and North Africa in relation to the treatment of disease." (*N.B.*—After the meeting the members of the Section will dine at Pagani's Restaurant, 42 Great Portland Street, W., at 7 p.m. Price of tickets [exclusive of wine] 3s. 6d. Members may bring guests. The secretaries would be glad to receive names of members intending to dine.)

At 5 p.m.—**April 21, Dermatological Section:**
At 11 Chandos Street, W.
*Cases*: Mr. J. E. R. MacDonagh: (1) Dermatitis herpetiformis. (2) Some cases of syphilis illustrating the importance of Wassermann's reaction.

At 4.30 p.m.—**April 22, Section for the Study of Disease in Children:**
At 11 Chandos Street, W.
*Cases*: Dr. F. Langmead: Case of congenital scoliosis due to spinal malformation. Dr. L. G. Parsons: Case of congenital acholuric jaundice. Dr. R. Hutchison: (1) Two cases of oxycephaly. (2) A case of speech defect. And other cases.
*Paper*: Dr. F. Langmead: A short paper on eighty consecutive cases of wasting infants fed on undiluted citrated milk.

At 8.30 p.m.—**Epidemiological Section:**
At 15 Cavendish Square, W.
*Paper*: Dr. Sheridan Délépine: "Contribution to the study of the influences determining the prevalence of bovine tuberculous mastitis."

ANNOUNCEMENTS.—Odontological Section: At the next meeting, on April 25, at 8 p.m.: *Paper*: Mr. A. Hopewell Smith: "Two odontoceles and some other cysts."
*Casual communications.*—Mr. C. Schelling: (a) "Two cases of suppuration in the maxilla, and (b) a case of neuralgia due to impacted lower wisdom teeth, in all of which skiagrams helped the diagnosis." Mr. Herbert Tilley: "Two cases of suppurating dental cysts invading the antrum." Mr. W. Rushton: "A case of painful attrition." Surgical Section: The next two meetings will be held at the Botanical Theatre at University College, on Tuesday, May 10, and Tuesday, June 14, at 5.30 p.m., when a discussion on "The Present Position and Treatment of Syphilis" will be opened by Mr. Ernest Lane. Sir Jonathan Hutchinson, Professor Fleming, and Mr. Arthur Shillitoe are amongst those who have promised to take part. Gentlemen wishing to speak in the discussion are requested to send their names as soon as possible to the Hon. Secretary, Mr. J. Hutchinson, 1 Park Crescent, W.

According to the *American Critic and Guide*, the Pathology Club gave its annual dinner in the gallery of the Hepatic Clubhouse. About sixty guests assembled, with Pellagra, master of ceremonies, seated upon a throne of cholesterin crystals between a hook-worm, wearing a girdle of staphylococci, and a Cohnheim cell with a metastasis license conspicuously displayed. Pellagra wore a decoration, conferred by the Pathology Academy, for valorous service at Andersonville prison during the Civil War. The guests were seated at an elevated cicatrix which served as a table and marked the site of an old typhoid ulcer in the gall bladder. The diphtheria germ received the Pellagra gold medal for the best costume worn by a club member. He wore his famous side-chain robe, woven out of amboceptors and complements. The guests' medal was awarded to the colon bacillus, who drove a pair of trained amœbæ around the banquet hall. A one-act tragedy was enacted in which the valorous but ineffectual resistance of the plasmodia family to their ancient enemy, quinine, was graphically depicted. It reduced the audience to karyokinetic evidences of grief. This was followed by a short farce, in which was represented the hazing and eviction of Beard's pancreatic ferments. Cancer roared and laughed loudly at the absurd performance. The sleeping sickness organism, between yawns, then moved an adjournment and the party broke up, soon disappearing down the portal stream. The menu was as follows: Ptomaine cocktails; serum, plain; muscæ volitantes; small round cells; bundle of his, vitreous sauce; pineal croquettes; cilia; phagocyte punch; agar-agar pudding, albumin sauce; opsonic ices, all strains; lady fingers; casein nodes; caffein cordial.

Mr. James Paterson, of Midlothian, has left £40,000 for the foundation and maintenance of a nursing home in the Bridgeton district of Glasgow, and £5,000 to each of four Glasgow institutions, and £20,000 for such charitable purposes as the trustees may select.

For Hospitals and Institutions.

| | |
|---|---|
| Analysis Ledger. 3 vols. (Uniform with the new Index of Classification adopted by the King's Hospital Fund) ... ... ... ... | £1 12s. 6d. |
| Cash Book ... ... ... ... ... ... ... | 12s. 9d. |
| Cash Analysis and Receipt Book ... ... ... | 16s. 6d. |
| Secretary's Petty Cash Book ... ... ... | 13s. 0d. |
| List or Register of Annual Subscribers ... ... | £1 0s. 0d. |
| Linen Register ... ... ... ... ... ... | 7s. 6d. |

And many other Ledgers necessary in the office of large and small institutions.

Of all booksellers or of The Scientific Press, Limited, 28 and 29 Southampton Street, Strand, London, W.C.

### Jottings.

A new convalescent home in connection with the Belfast Hospital for Sick Children has been obtained in Carrickfergus, and was opened on April 2 by Mrs. Robinson, the wife of the Dean of Belfast.

Considering the size of Forest Row, Sussex, and the relatively small number of working-men in it, the village may be congratulated on having raised, after all expenses had been paid, £23 12s. by its Working Men's Hospital Committee. The money is divided among the hospitals at which the members have received treatment.

The Lord Mayor of Manchester, Mr. Councillor Behrens, has led the crusade on behalf of Ancoats Hospital, which needs £20,000 for the endowment fund. The Lord Mayor has not only supported the appeal by his publicly expressed sympathy with the hospital, but has backed this up by the gift of £100.

The Leicester Private Fire Brigades' Association (Sir Samuel Faire, President) is holding a National Tournament for the Private Fire Brigade Championship of all England on Saturday, June 18, when the whole of the profits—less bare expeneses—will be generously voted by the Committee to the funds of the Leicester Infirmary Children's Hospital.

St. Peter's Hospital for Stone, etc., Henrietta Street, Covent Garden, W.C., is celebrating its jubilee by a festival dinner at the Ritz Hotel, Piccadilly, W., on Tuesday, April 26, at which the Right Hon. Lord Airedale, P.C., will preside. The Secretary (Mr. Irwin H. Beattie) will be pleased to receive the names of gentlemen willing to act as stewards, or to acknowledge contributions (however small) towards the sum required to clear off the debt and to provide for the upkeep.

The annual meeting of the Hospital Saturday Fund, over which Sir Savile Crossley presided, took place last week. The receipts for the year showed a gratifying advance of about £800 on those recorded in 1908, reaching the total of £30,662. The management expenses also had declined, and were only 7.9 of the gross moneys received. Hospitals who benefited by the Fund numbered 213, including convalescent homes, and the amount distributed reached £28,471.

The amount already contributed to the Extension Fund of the Norfolk and Norwich Hospital is announced to be just over £18,000; and it is hoped that the first of the new buildings will be finished by October next. But it is pointed out that a much larger sum is still urgently needed to carry out the full scheme, which is the union of the Eye Infirmary with the Hospital, and the arrangements for this are waiting the collection of the necessary sum for erecting a building.

The question of hospital abuse has been raised at the Wolverhampton and Midland Eye Infirmary. Mr. T. Capper said that Dr. Chesshire had shown that people were coming to the infirmary who could afford to pay, and he was sorry to say that many poor people who could not afford to pay had been shut out. The abuse apparently arose from the careless way in which hospital notes were distributed. Mr. Capper insisted that those who had tickets to dispose of must really make some show of discretion. It should be added that Mr. Capper made it clear that Dr. Chesshire's remarks did not apply to working-class families, but to another class which it would be inconvenient to mention. It was at a meeting of the Hospital Saturday Committee of the institution that the above statements were made.

The Princess of Wales has sent a copy of Burdett's "Hospitals and Charities" to the Invalid Children's Aid Association (London), incorporated, of which she is the patron.

The annual general meeting of the National Association for the Establishment and Maintenance of Sanatoria for Workers suffering from Tuberculosis will be held on Wednesday, April 20, at the offices, 52 Gray's Inn Road, London, W.C., at 8 p.m.

The new private-ward of the Hawick Cottage Hospital and Dispensary, Glasgow, is nearly finished. Its beginning was due to the generous gift of the trustees of the late Mr. Sheriff Anderson, who subscribed £500, which will also pay for the cost of furnishing and for a new nurse's room. It will be christened the "Anderson Ward."

As the result of a meeting of the Midland Railway men at Bradford, which was addressed by Mr. David Wade, the chairman of the Hospital Fund, and by Mr. Herbert Gill, the honorary secretary, a systematic collection for the hospital funds was decided upon unanimously. The Bradford draymen have also decided to start a similar fund.

Beneath the signatures of the following gentlemen a letter has appeared in newspapers in support of the appeal for £10,000 for the maintenance of University College Hospital. The letter says it has been "definitely decided to close several wards on June 1 next unless adequate help is forthcoming before June next." Bedford (President), Henry Lucas (Chairman and Treasurer), Walter Baily (Vice-Chairman), Thomas Barlow, M.D., F.R.S., Arthur E. Barker, F.R.C.S., H. Batty Shaw, M.D., F.R.C.P. (Dean).

### Gifts and Bequests.

Mr. Edward Elsey, lace manufacturer, has left £1,000 to the Nottingham General Hospital.

The late Mr. Edward Thomas Hutchinson, of Leicester, has bequeathed £100 to the Leicester Infirmary.

The Clothworkers' Company has sent £100 to the Royal Hospital for Diseases of the Chest, City Road, E.C.

Mr. Jacobus Bernardus Gelder, of Stroud Green, N., has left £1,000 to the Great Northern Central Hospital, Holloway Road, N. Subject to a life interest in a sum of £6,000 and the payment of the income from £2,000 for the benefit of a nephew until he shall attain his majority, the sum of £6,000 is to revert to the Great Northern Central Hospital.

Mrs. Martha Sophia Webb, of Great Gransden, Hunts, has left £400 to Addenbrooke's Hospital, Cambridge, and £100 to the Missions to Lepers.

Mr. George Pick Smith has left sums estimated at £10,000 to be divided into four equal parts, and distributed amongst the following institutions: One-fourth to the Bolingbroke Hospital, Wandsworth Common; one-fourth to the Free Hospital for Children of the Poor, "Kingsholm," Gloucester; and one-fourth between the General Infirmary, Gloucester, and the Gloucester Eye Institution, Southgate Street, Gloucester.

Mr. Reuben James Robinson, of Ashbourne, Derbyshire, retired farmer and horse dealer, probate of whose will has been granted to Dr. Alexander Boswell, M.D., of Church Street, Ashbourne, has left to his medical attendant, Dr. Alexander Boswell, £2,250, and the residue of his property, apparently about £5,000, to the Manchester Royal Infirmary and Dispensary.

### THE QUESTION BOX.

**SPECIAL NOTICE.**—Questions of interest affecting the honorary medical staff and hospital administration in all departments are invited. When any point arises we hope our readers will formulate it in a question and so co-operate to make this column of real value and interest. In this way the experience of the best-managed hospitals should be made helpful to the whole hospital world. We shall welcome the contribution of both questions and answers.

N.B.—The publication of an answer in this column does not necessarily preclude the publication of subsequent answers to the same question, for a question may involve points which require to be threshed out and fully discussed. Answers, if published, will be paid for at scale rates.

### THE PURCHASE AND CONTROL OF PROVISIONS.

Question: Is it in the best interests of an institution for all provisions, etc., to be purchased by, and to be under the direct control of, the matron's or the secretary's department?

### A Manchester View.

By Mr. W. G. Carnt, General Superintendent and Secretary, Manchester Royal Infirmary.

The answer to this question is one of degree. In a small hospital where the Secretary does not give his whole time to the service of the Institution, the duty of purchasing provisions should undoubtedly be left to the matron. The Secretary would probably be a local accountant who would not be likely to have received the special training which alone could qualify him to carry out this duty satisfactorily, and, moreover, as provisions have to be ordered from day to day, it would be impossible for him to accept the responsibility. In a hospital sufficiently large to employ the full time of the Secretary, who should also be the Superintendent, all orders, whether for provisions or anything else, should be signed and despatched to the tradesmen by him, as he, as the executive officer of the Board of Management, should alone be authorised to pledge the credit of the Institution.

The actual custody of the provisions in a hospital of moderate size, say, from 100 to 200 beds, should be undertaken by the Matron's Department, her requisitions for food being sent to the Secretary's office for order at a fixed hour daily. In a hospital of 100 beds the matron and her assistant could keep the provision store and the comparatively simple provision stock-books without much trouble, but in a hospital of larger size she should have the assistance of a skilled housekeeper—not a nurse, but a lady who has devoted herself to housekeeping and is likely to remain some years in the institution. The evil of the trained nurse housekeeper is that she looks upon her post as a stepping-stone to a matronship. She is always applying for vacancies in other hospitals, and when she is just beginning to be of value she obtains the matronship she covets and all the work of training a housekeeper has to be undertaken again.

It should be the duty of the Secretary to inspect meat, fish, and milk daily, although the actual weighing and measuring should be done by the housekeeper, and it should be the duty of the matron and the housekeeper to call his attention to any other provision supplies which do not appear to them to be satisfactory. It is perhaps a question whether in hospitals of from 150 to 200 beds it would not be an advantage for the housekeeper to be responsible directly to the Secretary for the control of provisions.

In a hospital above 200 beds the matron should certainly be relieved entirely from this responsibility. The provision and all other stores should be in the charge of a storekeeper or steward who should be responsible directly to the Secretary. More elaborate books of account are necessary, there are more wards, and greater supervision of issues must be exercised so as to ascertain the cost of provisions in each ward, and for each department of the household, to enable the Hospital Committee to keep a check upon expenditure, and this work is better done by a storekeeper under the direct supervision of the superintendent and Secretary than in any other way. The storekeeper's requisitions for provisions must, of course, be submitted daily to the Secretary for order.

### THE STAFF OF A COTTAGE HOSPITAL.

Question: What staff is required or usual in a cottage hospital of 16 beds, consisting of two private wards and four wards with three beds each, all on ground floor; laundry done in the hospital?

### Answer.

For a hospital containing 16 beds, four of which are in private wards, a staff of two trained nurses and two probationers would probably be required. Much, however, depends on whether the beds are constantly filled, and with what class of case. If the daily average of occupied beds were no more than nine or ten, and some of the cases were chronic or convalescent, one probationer ought to be sufficient. One nurse would be on night duty, and the other, with one probationer, would be able to manage, but in that case the matron would be obliged herself to take regular turns on duty in the wards.

For the domestic staff, one cook, one housemaid for the staff, and one ward maid or scrubber for the wards ought to be sufficient. One laundry maid would probably suffice, giving all her time to the work, and it would be cheaper in the end to pay for labour-saving apparatus in the laundry.

Until the institution has been in working order for six months, the matron should guard herself against any definite decision about the number, either of nursing or domestic staff. Let it be understood that she is making trial with a view to reducing the numbers as far as is consistent with efficiency, and then let her make detailed report to her committee when she has satisfied herself what assistants she needs.

### OUT-PATIENT CASUALTY ADMITTANCES.

Question: Should accidents admitted as in-patients from the casualty department be counted as casualties?

---

Recently, in the course of an address at a fête in aid of the Consumptive Homes at Sydney, Sir Philip Sydney Jones, M.D., pointed out that the annual loss to the Commonwealth is 4,000 persons, and that during the last 33 years New South Wales has lost 42,912 persons from consumption. Something may be done when the various Governments and Boards of Public Health have agreed upon effectual methods of prevention and treatment. So far the most sensible suggestions seem to have come from Dr. Alex. Pentland, of Singleton, N.S.W., who points out how easy it would be in this large country with its small population to find open-air spaces on coast or inland for camps or farms with all the *desiderata* of germ-free air, absence of all sources of infection, cool nights in summer, and freedom from dust.

After four years, Professor R. J. A. Berry, who came to Melbourne from Edinburgh, has gone on leave of absence to visit Europe. Professor Berry is the first Melbourne professor of anatomy, and thus has had to create a new department. He has shown a stimulating interest in Australian ethnology.

The new Bovril picture scheme, by which an engraving of "The Vicar of Wakefield" or "The King's Derby, 1909," by Mr. W. Hatherell, will be given in return for coupons of the face value of one guinea, is now complete. Up to June 30, 1910, every bottle of Bovril will contain a coupon, the value of which varies in proportion to the size of the bottle. The engravings are sure to be popular, as the pictures already are from which they have been reproduced.

THE HOSPITAL. April 16, 1910.

*Name* ..............................

*Address* ..............................

..............................

This Coupon must accompany manuscript or contributions intended for The Hospital.

**THE MEDICAL LIBRARY.**

Post Free.

"Medical History: From the Earliest Times." By E. T. Withington, M.A., M.B. Oxon ... 12s. 6d.

"The Consumptive Working Man. What Can the Sanatoria Do for Him?" By Noel D. Bardswell, M.D. ... ... ... ... ... ... 10s. 10d.

"Photomicrography." By Edmund Spitta, M.R.C.S. With 41 Half-tone Reproductions from original negatives and other Illustrations ... ... 12s. 0d.

Of all booksellers or of The Scientific Press, Limited, 28 and 29 Southampton Street, Strand, London, W.C.

